# Magnetic Force Microscopy of Micropatterned Clusters of Superparamagnetic Iron Oxide Nanoparticles

**DOI:** 10.1021/acsanm.5c01383

**Published:** 2025-06-05

**Authors:** Kenzington L. Kottenbrock, Sierra Reis, Gunjan Agarwal, Samuel D. Oberdick

**Affiliations:** † Biomedical Engineering Graduate Program, 2647 The Ohio State University, Columbus, Ohio 43210, United States; ‡ Department of Mechanical and Aerospace Engineering, The Ohio State University, Columbus, Ohio 43210, United States; § Department of Physics, University of Colorado, Boulder, Colorado 80309, United States; ∥ 10833 National Institute of Standards and Technology, Boulder, Colorado 80305, United States; 5 Department of Physics, The Ohio State University, Columbus, Ohio 43210, United States

**Keywords:** magnetic force microscopy, magnetic nanoparticles, nanomaterials, scanning probe microscopy

## Abstract

Magnetic force microscopy (MFM) was used to characterize micropatterned clusters of superparamagnetic iron oxide nanoparticles (SPIONs). Top-down lithography was used to create SPION aggregates with well-defined geometries. The micrometer-scale aggregates exhibited different properties from individual particles and from smaller clusters containing just a few particles. The MFM phase shift from magnetic interactions between the sample and probe tip could be detected at lift heights of several hundred nanometers. The experimental data was compared to a magnetic dipole–dipole interaction model to understand the relationship between MFM phase shift and lift height. Magnetic interactions between the probe tip and the sample also led to an apparent “ballooning” of the feature size, where the aggregates appeared larger with MFM than their physical size obtained from scanning electron microscopy. These results can guide emerging applications of MFM, such as the detection of SPIONs within biological environments.

## Graphical abstract

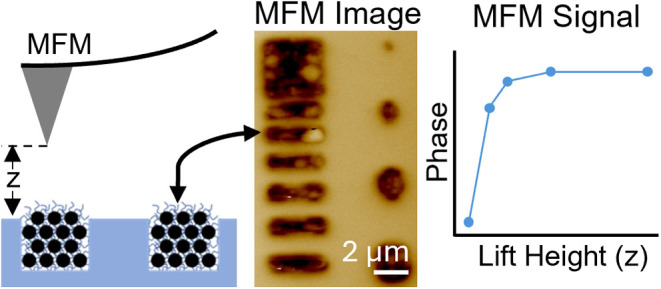

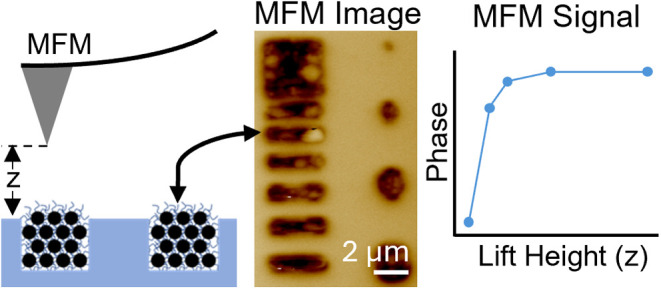

## Introduction

Superparamagnetic iron oxide nanoparticles (SPIONs) are of increasing interest in the fields of biology and medicine for applications such as cell sorting,^1,2^ drug delivery,^3–5^ hyperthermia,^6–9^ and as contrast agents in magnetic resonance imaging.^10,11^ At low magnetic fields, SPIONs have very low to zero remanent magnetization and a high magnetic susceptibility. As magnetic fields increase, these materials show an increase in magnetization followed by an approach to saturation at high fields. Magnetometry is often used to measure many particles at once.^12,13^ These measurements provide average physical properties from a large ensemble of magnetic particles. The behavior of individual SPIONs has been explored using high-resolution experimental techniques, like magnetic force microscopy (MFM).^14,15^ On scales between large ensembles and individual particles, SPIONs can form aggregates that exhibit complex magnetic behavior. These mesoscale clusters are especially relevant for biological applications, since SPIONs can aggregate and pack together to form mesoscopic structures in cells and tissue.^16,17^ These aggregates can have different magnetic properties compared to an ensemble of homogeneously dispersed, weakly interacting particles.

High resolution microscopy approaches like MFM can serve as a valuable tool for characterizing SPION aggregates. MFM is a technique based on atomic force microscopy (AFM) that uses a magnetically coated probe to measure topography and long-range magnetic forces from a sample with the characteristically high lateral resolution of AFM. It has historically been used to study magnetic storage media^18–20^ and other ferromagnetic materials^21^ but has recently grown in popularity to study SPIONs both in vitro and in biological systems, as mentioned in the review by Kazakova et al.^22^ MFM has been previously used to evaluate a variety of magnetic properties such as magnetic anisotropy^23,24^ and the magnetic moment of individual magnetic nanoparticles in an applied magnetic field.^25^ Limited studies exist on MFM analysis of magnetic nanoparticle assemblies, but there has been promising evidence of MFM’s usefulness in this line of research.^26–29^ In a study conducted by Puntes et al.,^27^ two-dimensional self-assemblies of cobalt nanoparticles showed density dependent behavior when imaged with MFM. High density areas behaved like ferromagnetic thin films with correlated in-plane magnetization across several particles (similar to domains), due to dipole interactions. Conversely, dispersed noninteracting nanoparticle islands behaved as single dipoles. Iacovita et al.^28^ presented MFM measurements on iron oxide nanoparticle clusters under the influence of an external magnetic field. They observed a discrepancy between the vibrating sample magnetometry (VSM) and MFM data. VSM measurements on the powder samples exhibited ferromagnetic behavior. However, MFM measurements of individual clusters showed superparamagnetic behavior. The authors speculated that the clusters formed large aggregates. When exposed to an external magnetic field, dipolar interactions between the aggregates stabilized the magnetization in the powder, leading to the ferromagnetic behavior. These studies demonstrate the usefulness of MFM in the study of magnetic nanoparticle assemblies. The studies also show that the magnetic behavior of these assemblies is very complex, since the net MFM signal detected from SPION assemblies is a result of the collective magnetization of many nanoparticles. To better understand the MFM signal from nanoparticle assemblies, new experimental systems are needed with precise control over aggregate size, shape, and spacing.

In this study, SPION aggregates with well-defined geometrical patterns were created using microfabricated templates. The top-down microfabrication of SPION clusters enabled quantitative comparison between MFM signal and structural properties of aggregates. This technique provided a novel means to create large clusters (>1 μm) while still maintaining control over their size, shape and spacing. MFM was also used to measure the properties of micropatterned iron thin films, for comparison with SPION samples. The SPION aggregates showed a stronger MFM phase signal than spin-coated particles, with a discernible signal at lift heights of several hundred nanometers. The data could be fit using a dipole–dipole interaction model, where the probe tip and a subvolume of the SPION sample were both treated as magnetic dipoles. Finally, we compared the apparent feature size of SPIONs and micropatterned iron thin films using several microscopy techniques, and found that the sizes measured with MFM could appear larger than actual size due to probe-sample interactions.

This research was motivated by emerging applications of MFM, specifically detection of magnetic particles in biological environments. SPIONs can form aggregates with a broad distribution of shapes and sizes in biological environments, creating complications for quantitative MFM.^30^ In this study, top-down microfabrication was used to create geometrically precise clusters of SPIONs to better understand the relationships between cluster geometry and quantitative features of the MFM image, such as phase signal and resolution. These experiments can be used to guide future work on magnetic particles embedded within biological matrices.

## Materials and Methods

### Synthesis of SPIONs

SPIONs were synthesized using a procedure adapted from Sun and Zeng.^31^ All chemicals were acquired from Sigma-Aldrich unless otherwise specified. Oleic acid (1.70 g), oleylamine (1.61 g), iron­(III) acetylacetonate (0.71 g), 1,2-hexadecanediol (2.58 g), benzyl ether (15 mL), and a magnetic stir bar were added to a 125 mL three neck flask. The flask was sealed and pumped/purged three times with nitrogen gas. Then, the solution was heated under nitrogen gas at 200 °C for 2 h before being raised to 298 °C for 1.5 h. After the synthesis, the reaction products were washed twice using a mixture of 1:1 acetone and ethanol, and by centrifuging the products at 6000 rpm. Finally, the nanoparticles were resuspended in toluene and stored in a sealed glass scintillation vial for future use.

### Transmission Electron Microscopy

The stock nanoparticle solution was diluted by a factor of 50 in toluene and added dropwise to the surface of a Formvar/carbon-coated transmission electron microscopy (TEM) grid (Ted Pella) to prepare samples for imaging. TEM was performed using a Tecnai T12 Spirit BT microscope with a LaB_6_ filament operating at a voltage of 100 kV. ImageJ software was used to analyze the TEM images for measurement of the average diameter of nanoparticles.^32^

### Magnetometry

A Lake Shore 8607 vibrating sample magnetometer (VSM) was used to characterize diluted SPIONs and clustered SPIONs. To prepare the diluted sample, the SPIONs were mixed with a photocurable polymer (0.6 mg Fe per mL) and the sample was prepared by curing the composite with ultraviolet light (details in Supporting Information). To prepare the clustered sample, the SPIONs were drop cast on top of a silicon wafer. After evaporation, the SPIONs formed large aggregates on the surface. The VSM magnetometry was performed at ambient laboratory room temperature. Data was collected from 2 T to −2 T.

A Quantum Design MPMS 3 superconducting quantum interference device (SQUID) magnetometer was also used to characterize the dilute sample. The magnetic moment was measured as a function of applied field from 7 T to −7 T at 300 K. The high field measurements were used to determine the saturation magnetization of the particles (Supporting Information).

### Fabrication of Silicon Wells Filled with SPIONs

Silicon wells were created using photolithography and etching. Silicon wafers, photoresist, developers, and other chemicals were supplied by the Microfabrication Facility at the National Institute of Standards and Technology, Boulder. Silicon wafers were first cleaned with solvents (isopropanol and acetone), and then exposed to an oxygen plasma for additional cleaning. Then, an adhesion promoter and a layer of photoresist (Megaposit SPR 660, 2600 rpm spin speed) were spin-coated onto the wafer. The photoresist-coated substrate was baked at 95 °C for 60 s and thereafter exposed using an ASML 5500/100D wafer stepper with a dose of 250 mJ/cm^2^. Following exposure, the substrate was baked at 110 °C for 60 s. The photoresist patterns were developed by immersing the substrate in a developer based on tetramethylammonium hydroxide (TMAH) (MF 26 A, Microchem/Kayaku) for 30 to 45 s. After development, the wells were etched into the silicon wafer using deep reactive ion etching (DRIE) with a multiplex inductively coupled plasma (ICP) etching system from Surface Technology Systems (STS). Following the etch, the photoresist was removed using acetone and the wafer was cleaned using an O_2_ plasma (60 W, 50 sccm) for 5 min.

To fill the wells with SPIONs, a solution containing SPIONs in toluene was drop cast on the surface of the micropatterned silicon. Approximately 20 to 30 μL of solution was pipetted on top of the silicon during a standard procedure, which was enough to coat about 1 cm^2^ of the substrate. Then, the solution was allowed to dry in a fume hood. After evaporation, the substrate was further dried using compressed N_2_ gas and a hot plate (70 °C for 3 min 30 s). This produced an inhomogeneous layer of nanoparticles across the surface and within the silicon wells. The evaporative coating procedure was performed 3 times to ensure that the silicon wells were entirely filled with SPIONs. Then, the surface of the SPION-coated chip was polished using the rough, unpolished side of another silicon wafer for several minutes, which removed the particles from the surface, but left SPIONs within the wells.

The micropatterns were repeated many times in dense arrays across large areas of the substrate. Prior to experiments, several regions were profiled with AFM and MFM to identify patterns with relatively uniform filling (no large cracks or gaps). These regions were used for MFM experiments.

As a control, randomly dispersed SPIONs and their clusters were generated by spin coating of SPIONs on silicon surfaces.

### Micropatterned Iron Thin Film

Iron micropatterns were fabricated by evaporating iron on top of photoresist patterns, followed by a lift-off procedure where photoresist was removed. The photoresist patterns were created using the same process flow as the photoresist for the silicon wells. Once the photoresist was patterned and developed, the substrate was loaded into an electron beam evaporation system from AJA International. 50 nm of iron was evaporated onto the substrate at a rate of 0.1 nm/s. After metal deposition, the photoresist template was removed by sonicating the substrate in acetone for 2 min.

All patterned surfaces were characterized via scanning electron microscopy (SEM) imaging. SEM images were acquired using a Zeiss Sigma 300 microscope operating at a voltage of 5 kV to 10 kV.

### Magnetic Force Microscopy

Magnetic force microscopy (MFM) was performed using the Bruker BioScope Resolve AFM in ambient air. Samples were adhered to metal disks, which were mounted on the AFM sample stage. High-moment MFM probes (ASYMFM-HM, approximate coercivity: 575 Oe, Asylum Research) or nonmagnetic AFM probes (HQ/NSC15/AI BS, Mikromasch) were used at a scan speed of 2 Hz and 256 lines per scan direction. The MFM probes were magnetized using a permanent magnet. After identifying the desired patterned regions in topographic images, MFM data (phase images) were acquired in lift mode at various lift heights *z* = 60 nm to *z* = 600 nm, using the “Lift by Sensor” mode.

Quantitative analysis was performed to evaluate the signal strength of the MFM phase signal using the NanoScope Analysis 2.0 software. For each bar-shaped pattern, a region of interest (ROI) of 3.98 μm × 0.86 μm within the bar was selected. The average phase and its maximum phase magnitude were recorded. In some samples, artifacts appeared for lift heights of 60 nm and 100 nm, which were some of the lower lift heights used to characterize the micropatterned samples. In these cases, an ROI of 2.03 μm × 0.23 μm was used to avoid artifact interference. The area of negative phase of the patterned regions was ascertained using the “Particle Analysis” tool. For the cluster proximity analysis, an analysis of variance (ANOVA) and Tukey’s Post Hoc test were used to assess statistically significant phase differences across the clusters. A *p*-value of <0.05 was considered to be statistically significant.

## Results (with Discussion)

### MFM Contrast and Interactions between the Probe Tip and Sample Materials

Figure 1 has a schematic to help the reader visualize the interactions between the MFM probe tip and the types of samples used in this study. An understanding of how MFM contrast is created by the interactions between the probe tip and sample is helpful for understanding the results.

**Figure 1:**
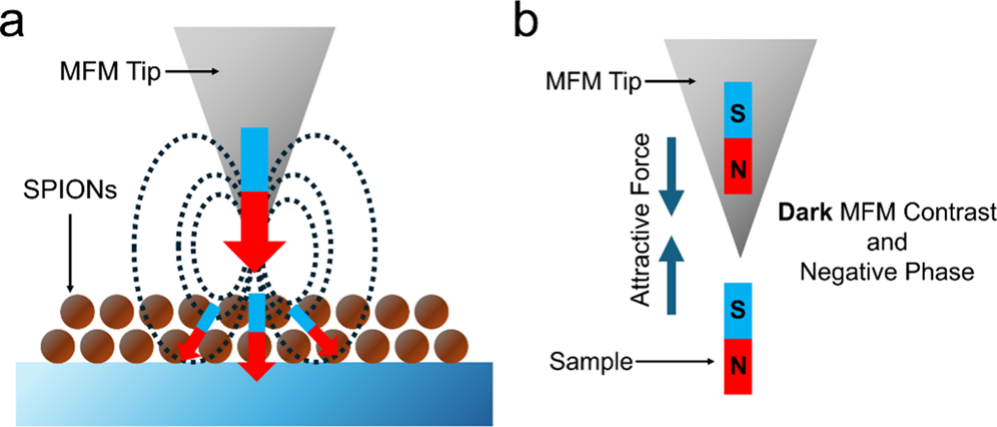
Diagrams displaying the interaction between the MFM tip and the sample. (a) The SPION sample is magnetized by the probe tip. (b) The induced magnetization in the samples creates an attractive force between the MFM probe tip and the sample, which results in a negative MFM phase shift and dark contrast in MFM images.

For this study, a high moment MFM tip was scanned across the surface of samples. As the MFM probe tip passed over the SPIONs, it induced an out-of-plane magnetization in the samples (Figure 1). The induced magnetization created an attractive force between the sample and the MFM tip. This creates a negative MFM phase shift and manifests itself as dark contrast on MFM images. The negative contrast caused by attractive interactions was most clearly observed at moderate to large lift heights.

At low lift heights, positive contrast could also be observed in the samples. Positive MFM phase shifts are caused by repulsive forces, like electrostatic repulsion. Positive contrast can also be caused by topographic cross-talk. To minimize the repulsive contrast and isolate the attractive magnetic interactions between the probe tip and sample, measurements were performed at relatively large lift heights.

### SPIONs Characterization

The SPIONs used in this study were characterized using magnetometry and TEM. Magnetometry was performed at room temperature on diluted and clustered samples of SPIONs (Figure 2a). Diluted samples were prepared by embedding SPIONs in a polymer matrix. The clustered samples were prepared by drop-casting nanoparticle solution on a nonpatterned silicon surface. After evaporation, the substrate was covered in SPION aggregates with random shapes and sizes. The random clusters were used for magnetometry, rather than the SPIONs in micropatterned wells, because they had a higher magnetic signal (due to a higher number of particles). The diluted samples show superparamagnetic behavior, where the sample has near-zero remanence at zero field, and a nonlinear approach to a saturated moment at high fields. The clustered particles, however, have a small but nonzero remanence at zero field and a slight coercivity (Inset of Figure 2a).

**Figure 2:**
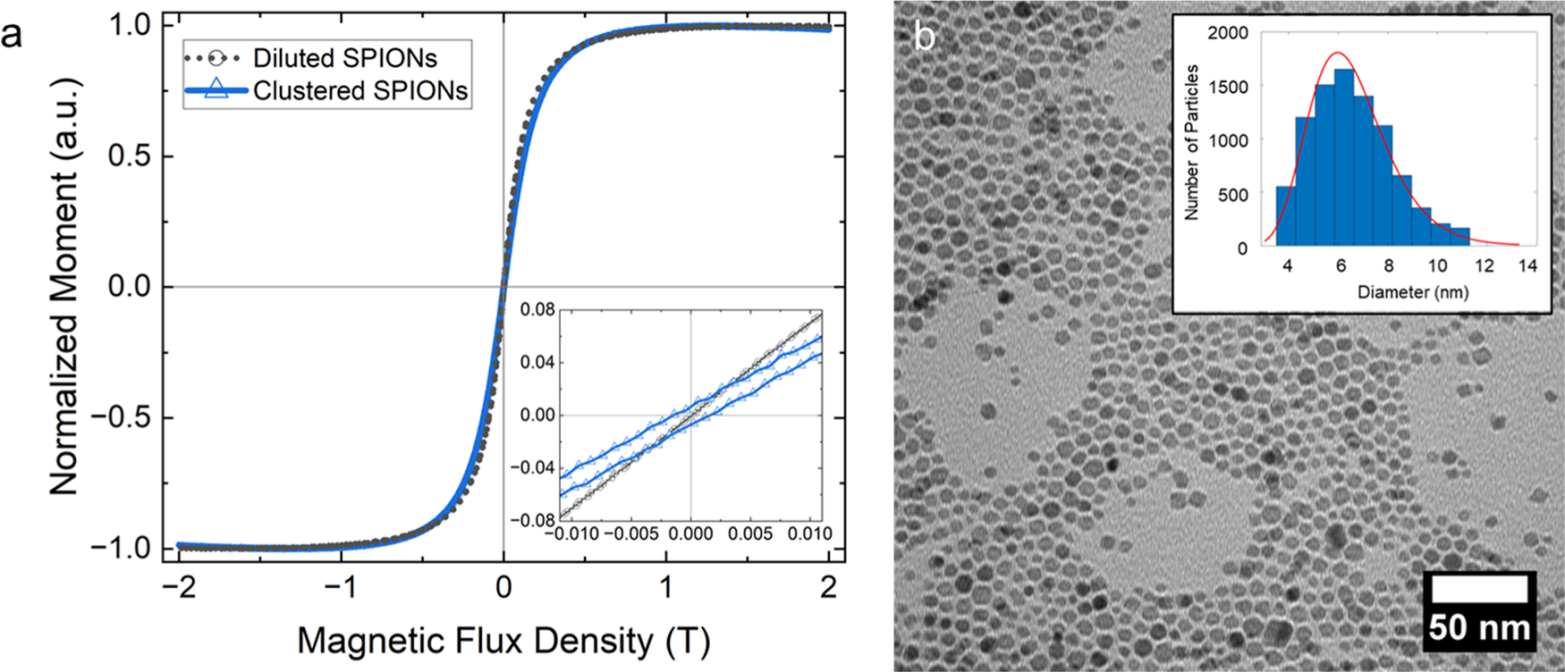
Characterization of SPIONs. (a) Normalized magnetic moment as a fuction of magnetic flux density for diluted and clustered SPIONs at room temperature. (b) Transmission electron microscope (TEM) image of the nanoparticles. Inset shows the size distribution of the particles. The particles had an average diameter of 7 nm ± 2 nm.

The TEM data was analyzed to estimate an iron oxide core size of 7 nm (Figure 2b).

### MFM of Randomly Dispersed SPIONs

Spin-coating was used to prepare a sample containing single SPIONs and small clusters of SPIONs for comparison to microfabricated SPION clusters. MFM imaging indicated a heterogeneous distribution of cluster sizes and shapes (∼20 nm ± 12 nm). A positive phase shift was observed low lift heights (below 60 nm), and was likely caused by topographical cross-talk. At a lift height of 100 nm the positive phase shift disappeared, and a small signal of negative phase shift could be seen from magnetic interactions between the MFM tip and clusters of SPIONs. This signal appeared to depend on the cluster size (Figure S1 in Supporting Information).

### Micropatterned SPION Aggregates

Figure 3a–d shows the microfabrication process flow for producing SPION aggregates with well-defined geometries. Wells were etched into silicon wafers, then overfilled with SPIONS, and, finally, the excess particles were removed to produce SPION-filled wells. SEM images were collected to confirm the resulting shape, size, and spacing of the patterns (Figure 3e,f). SEM also confirmed the presence of SPIONs within the patterned wells (Figure 3g). AFM measurements on unfilled wells showed that they had a depth of ∼600 nm. These patterns consisted of identical bars of increasing proximity and circles of varying sizes. The sample pattern enabled experimental investigation of the relationship between geometrical factors, such as feature size and proximity to other features, and MFM phase.

**Figure 3:**
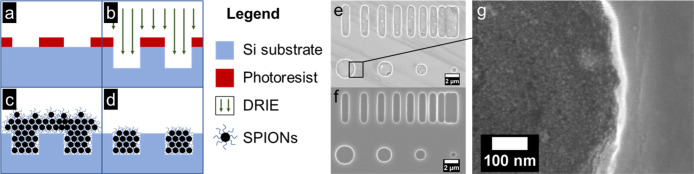
Schematic of the microfabrication procedure used to generate geometrically precise SPION aggregates: (a) photoresist is patterned, (b) deep reactive-ion etching (DRIE) is used to etch wells in the silicon substrate (∼600 nm deep), (c) the substrate is coated with SPIONs, and (d) lastly, the excess SPIONs are mechnically polished away. Representative scanning electron microscope (SEM) images of patterned wells with (e), (g) and without (f) SPIONs.

MFM imaging was performed on each sample. The samples were imaged using a nonmagnetic AFM probe to provide a control measurement. In the images collected, the empty wells showed areas of positive phase at the edges of the wells. The positive phase was especially apparent at low lift heights. The inner area displayed a slight negative phase even as the lift height extended past 200 nm. This was the case with both magnetic and nonmagnetic probes (Figure 4b–e and g–j). The SPION-filled wells showed distinctly different behavior. Using the MFM probe, the filled wells exhibited a large negative phase even at large lift heights (Figure 4l–o). When imaged with an AFM probe, the filled wells showed only small phase values in the positive or negative directions. Similar to the empty wells, some of the filled wells presented with areas of positive phase around the edges of the wells with both probes at a lift height of 60 nm (Figure 4l,q).

**Figure 4:**
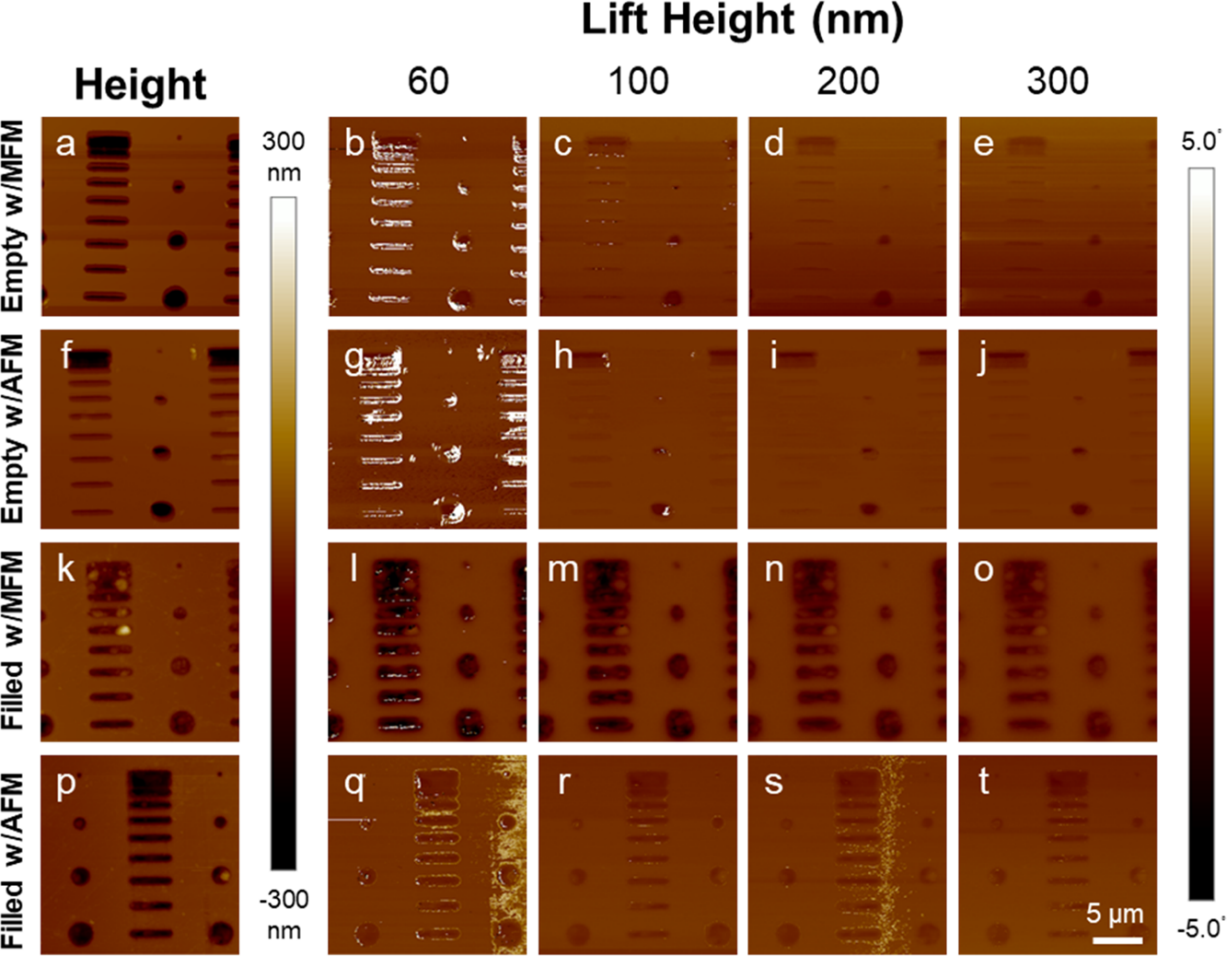
MFM analysis of patterned SPION aggregates. (a,f) Height images with MFM and AFM probes of empty control wells and (k,p) wells filled with ∼7 nm SPIONs. (b–e, g–j, l–o, q–t) Corresponding phase images at the indicated lift heights.

Micropatterned iron thin films with a layout matching the SPION-filled wells were also fabricated and measured with MFM (Figure S2). MFM measurements exhibited large negative phase values at large lift heights (Figure 5b–d). Areas of positive phase were present around the edges of the thin films at a 60 nm lift height with both MFM and AFM probes (Figure 5b,f). AFM measurements at large lift heights showed a decrease in the magnitude of the positive phase and no areas of negative phase (Figure 5f–h). With both probes, the phase shift (both positive and negative) decreased as lift height increased.

**Figure 5:**
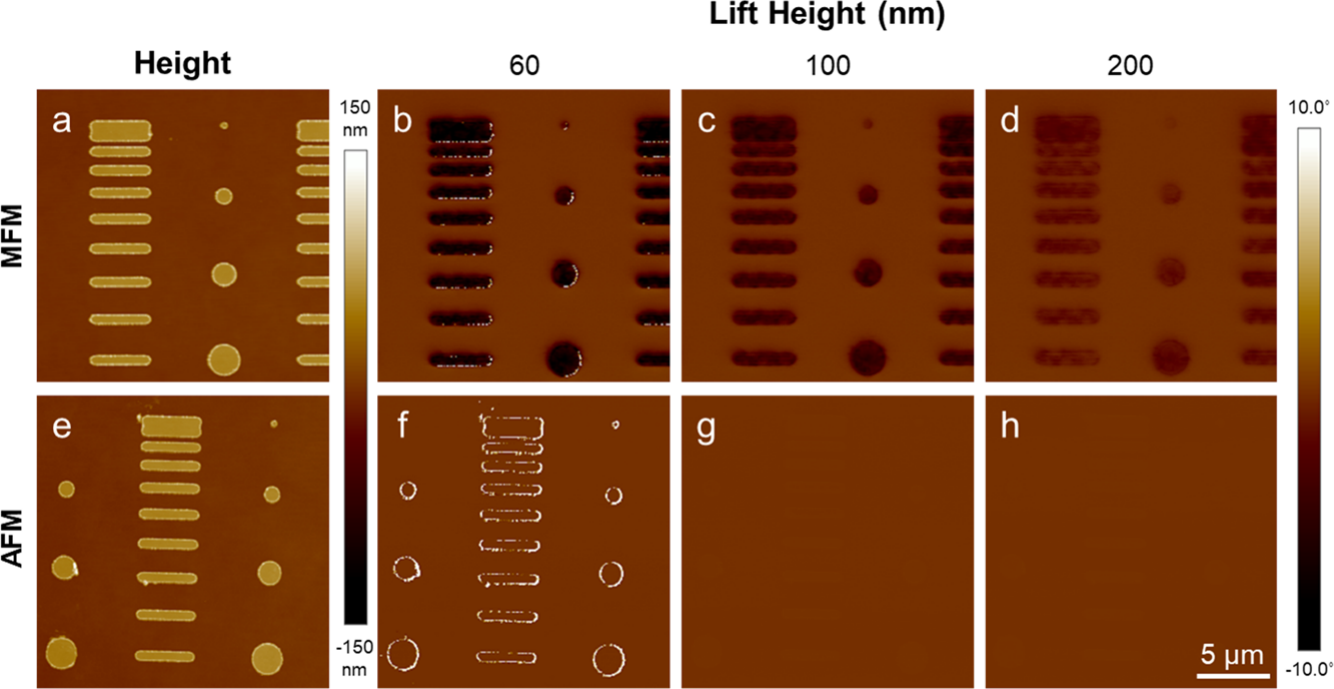
Height images of Fe thin films taken with (a) an MFM probe and (e) an AFM probe. (b–d and f–h) Corresponding phase images at the indicated lift heights.

For both samples, the magnitude of the negative phase shift decreased as a function of increasing lift height (Figure 6). The other combinations of samples (unfilled and filled) and probes (MFM and AFM) exhibited a negative phase value of comparatively low magnitude. The maximum negative phase at a lift height of 200 nm demonstrates that the MFM signal from iron thin films and SPION-filled wells can be clearly measured at lift heights of several hundred nanometers.

**Figure 6:**
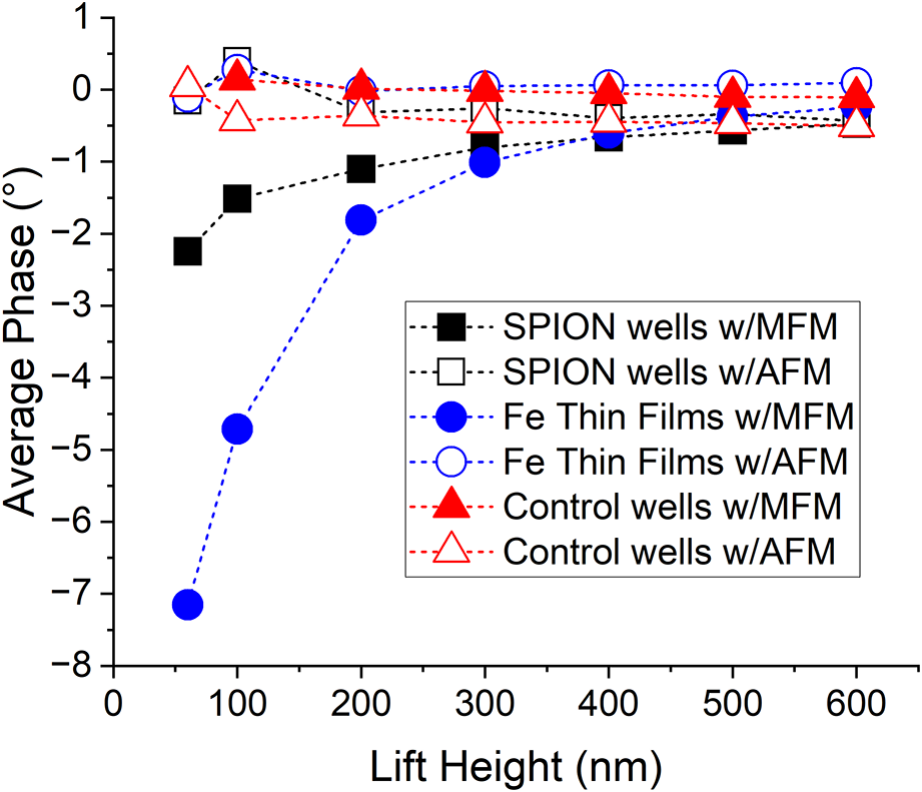
Experimental phase data as a function of lift height for each sample: empty wells, SPION-filled wells and Fe thin films. Each sample was imaged with an MFM and AFM probe.

Images were also analyzed to understand the relationship between lateral resolution and apparent feature size in MFM images. The round patterns displayed differences in apparent size depending on the imaging technique. The area of the round patterns was measured using SEM, MFM height (i.e., topography) and MFM phase images (Figure 7a–e). To measure the area of the clusters using MFM phase images, the area of phase below a threshold value was recorded. The MFM phase images perceived the objects to be larger than the measurements recorded using other methods. The measurements gathered from surface topography and SEM images were very similar. Regarding phase magnitude, a gradual decrease can be seen as the patterns increased in size. There was a significant difference between the average phase of the largest (∼2.5 μm) and smallest (∼0.5 μm) round SPION-filled wells. The two smallest iron thin films (∼0.5 μm and ∼1.5 μm) each had a phase that was significantly different from the rest of the round thin films (Figure 7f).

**Figure 7:**
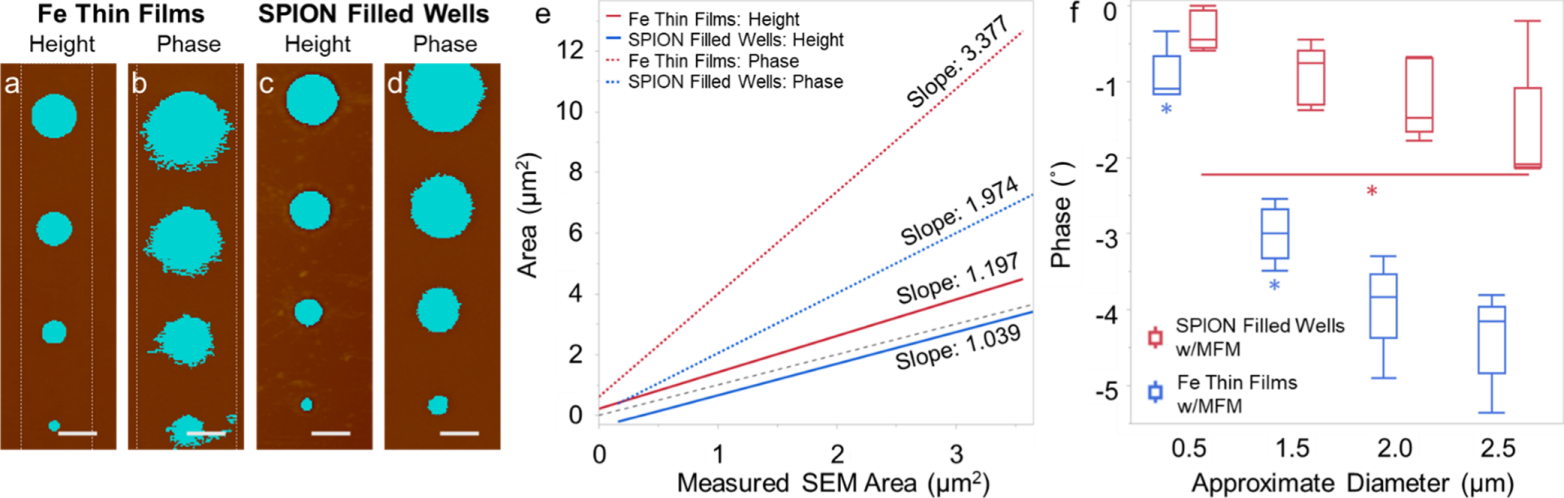
Analysis of round patterns. Multimodal assessment of cluster geometry: (a–d) thresholded height and phase images of the Fe thin films and SPION filled wells. Scale bar: 2 μm. (e) Line of best fit of the measured geometrical area of the circular patterns of SPION filled wells and Fe thin films using MFM phase images at a lift height of 100 nm and AFM topography (height) images. The *x*-axis is the measured geometry of the round clusters of each sample using SEM. The black dotted line has a slope of 1 and *y*-intercept of 0 for reference. All lines of best fit had an *R* ^2^ value ≥0.95. (f) Box plot of MFM phase as pattern size increases for SPION aggregates and iron thin films. Phase was measured at a lift height of 100 nm. The largest and smallest SPION filled wells had a significantly different phase from each other (*p* < 0.05). The two smallest Fe thin films had a significantly different phase from the rest of the Fe thin films (*p* < 0.05).

### Dipole–Dipole Model for Probe Tip-SPION Interactions

The experimental data for MFM phase as a function of lift height was fit using a simple dipole interaction model. The model treated the MFM tip and a subvolume of the overall SPION sample as magnetic point dipoles. Figure 8a shows a schematic of the model geometry and relevant parameters. The dipole moment of the tip was assumed to be at the center of the magnetic coating (100 nm thick CoCr for the tips used in the experiment) and spatially offset from the physical boundary of the tip. The dipole moment of the sample was treated as a subvolume of the overall sample, with a magnetic moment formed from the combined magnetization of many individual SPIONs.

**Figure 8:**
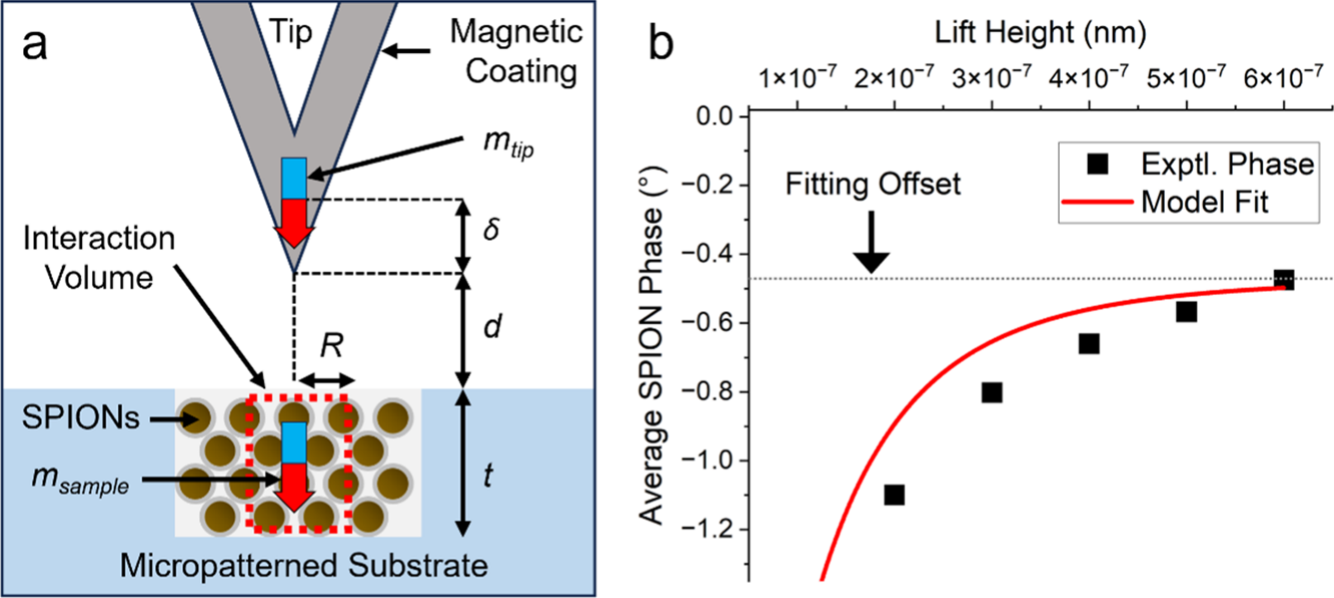
(a) Schematic showing geometry and parameters used for dipole–dipole interaction model. (b) Experimental phase data as a function of lift height with corresponding fit to dipole–dipole interaction model.

The MFM phase shift, Δφ, was calculated using the gradient of the force (magnetic force between the two magnetic dipoles) between the tip and the sample^22^

1 $$\Delta \varphi \left(degrees\right)=\frac{Q}{k}\frac{dF}{dz}=\frac{180}{\pi }\frac{Q}{k}\frac{6\mu_{0}m_{tip}m_{sample}}{{\left(\delta +\frac{t}{2}+d\right)}^{5}}$$

where *Q* is the quality factor of the MFM cantilever, *k* is the spring constant of the MFM cantilever, μ_0_ is the permeability of free space, *m*_tip_ is the magnetic moment of the tip, *m*_sample_ is the magnetic moment of the sample, δ is the distance of the MFM tip dipole moment from the surface of the MFM tip, *t* is the thickness of the interaction volume of the SPION sample, and *d* is the lift height. The model was fit to the experimental data using a cylindrical interaction volume, *V*, with a radius, *R*, and thickness, *t*

$$m_{sample}=MV=M\pi R^{2}t$$ 2

Figure 8b shows a fit of the model to experimental data. During the fit, all parameters were fixed except *R*. The thickness, *t*, was set equal to the thickness of the micropatterned wells (600 nm). Other parameters were chosen based on experimental details and material properties (Supporting Information). To exclude effects of topographic crosstalk, the fitting was limited to lift heights greater than to 100 nm. The model fit the data using *R* = 290 nm. This corresponded to a cylindrical interaction volume with a height of 600 nm and a diameter of 580 nm. Also, a constant offset was added to the fit to account for background signal. The offset, *C*, was chosen to match the phase at the largest lift height (600 nm).

## Discussion

The results of this study can be used to better understand how SPION aggregates respond to interrogation from MFM probes. The measurements of the spin-coated SPIONs and larger micropatterned SPION clusters both showed a uniform negative contrast at medium to large lift heights. This suggests that the MFM tip is inducing a magnetization within the particles by aligning their magnetic moments with the tip’s stray field (Figure 1a), which is consistent with other MFM measurements of SPION aggregates.^26^ There were differences, however, in the phase magnitude of the two samples. The spin-coated SPIONs, which had relatively small aggregates of particles (width <1 μm and height <50 nm), had a smaller phase signal than the larger micropatterned SPION clusters. The difference in phase signal was likely due to the larger magnetic volume of the micropatterned aggregates. The micropatterned wells were ∼600 nm deep and filled with SPIONs, whereas the spin-coated samples had regions populated with SPIONs that were, on average, 20 nm thick. These results suggest, somewhat intuitively, that larger clusters generated a larger, and more easily detected, MFM signal.

For biological applications, such as SPION detection in relatively thick tissue samples, it is important to understand the relationship between phase magnitude and lift height. The experimental data from this study was fit using a dipole-dipole model for magnetic interactions between the dipole moment of the tip and the induced dipole moment of the sample. Previous studies demonstrated that the MFM phase magnitude for single SPIONs followed a similar dipole–dipole interaction model.^33^ For the model used here, the induced dipole moment in the sample corresponded to a cylindrical interaction volume with a height of 600 nm and a diameter of 580 nm. This suggests that, during experiments, the MFM tip induced magnetization in many SPIONs and their collective magnetization combined to form the net phase signal.

The dipole–dipole interaction model used to fit the data was relatively simple. Still, it fit the data well and may serve as a useful guide for other MFM studies on SPION aggregates. In the future, more complex models or simulations (such as those that account for interparticle interactions and magnetic anisotropy) could be used to provide deeper insights into MFM measurements on SPION aggregates.

At low lift heights (≤100 nm), topographical cross-talk complicated the collection and analysis of experimental data. Topographical cross-talk can be caused by a variety of forces (e.g., van der Waals, electrostatic) and can cause either a positive or negative phase shift. In the micropatterned samples, cross-talk occurred at the sharp edges of the patterns. This was seen with MFM and AFM probes, indicating that this was not caused by magnetic forces. Negative phase shifts can also be seen in some control groups (Figure 4d,e,i,j,s,t). This phenomenon can have a significant impact on MFM studies because it can be difficult to distinguish topographical cross-talk from a phase shift caused by weak magnetic forces. In this study, though, the comparison of measurements performed with magnetic and nonmagnetic probes show that magnetic probes produce negative phase shift with considerably larger magnitudes than control groups (Figure 6a). This provides strong evidence that the negative phase shift is caused by the magnetic interaction between the sample and the magnetic probe.

While the SPIONs and iron thin films had different magnetic properties (superparamagnetic versus ferromagnetic), the iron thin films could be used to help validate MFM measurements. The iron thin films showed a consistent negative MFM contrast at large lift heights, indicating that the samples were being magnetized out-of-plane by the probe tip (Figure S6 in Supporting Information). This hypothesis was validated by magnetometry measurements of the patterned thin films (Figure S5 in Supporting Information), which indicated that the iron thin films had a larger magnetization than the SPION aggregates for fields and orientations relevant for MFM experiments. This was consistent with MFM measurements, which showed larger phase magnitude from the iron filmss.

MFM measurements on circular patterns with various sizes showed that MFM signal changed as a function of pattern diameter. The effect of pattern size on their phase shift was analyzed by assessing the phase of each of the round patterns at a lift height of 100 nm (Figure 7f). There was a significant difference between the average phase of the largest (∼2.5 μm) and smallest (∼0.5 μm) round SPION filled wells. This indicates that there is an increase in phase magnitude as the size of the wells increases. This can be partially explained using the results from the dipole–dipole interaction model. The smallest well (∼0.5 μm) had an overall volume that was similar to (or slightly smaller than) the induced interaction volume predicted by the model. The other three wells had volumes that were greater than the predicted induced interaction volume. Therefore, the maximum possible induced magnetic moment in the smallest features may have been limited by the physical dimensions of the wells, resulting in a lower signal than the larger features.

The micropatterned iron thin films also showed a size dependence. The two smallest iron thin films (∼0.5 μm and ∼1.5 μm) each had a phase that was significantly different from the rest of the round thin films. There was not a significant difference between the phase of the two largest iron thin films (∼2.0 μm and ∼2.5 μm), which may suggest that there is a nonlinear relationship between the phase and size of the iron thin films.

The experiments on circular features also showed that interactions between the MFM probe tip and the sample can influence the apparent size of sample features (Figure 7e). A comparison of SEM, AFM, and MFM images shows that the size measured by MFM is larger than the actual size of the features. This effect is caused by the interaction of the MFM probe tip with the stray field of the sample. The stray magnetic fields produced by features on the sample surface extend past their physical boundaries. As the MFM tip is scanned above the surface, it is sensitive to those fields and not the actual size of the feature. The net effect is “ballooning” effect in the MFM images, where the apparent size of magnetic features can be larger than the actual size. While previous studies have noted that the lateral resolution of MFM is determined by interactions between the tip and the sample,^34^ the measurements in this manuscript can be used to guide future experiments specific to SPION aggregates.

## Conclusions

Microfabricated arrays of SPION aggregates were characterized using MFM. Top-down creation of particle aggregates enabled measurement of MFM phase as a function of pattern geometry. The cluster size and lift height played a significant role in the phase shift measured by MFM. The data could be fit using a simple model based on dipole–dipole interactions between the magnetic moment of the probe tip and the induced magnetic moment in an interaction volume containing many SPIONs. Also, a “ballooning” effect was found in MFM phase images, where the feature size measured by MFM was larger than the physical size of the aggregate. These results can guide future MFM research on magnetic nanoparticles in biological systems. However, more work will be needed to understand how the complex nanomagnetism of particle aggregates affects quantitative MFM images.

## Supplementary Material



## Data Availability

The data that support the findings of this study are available from the corresponding author upon reasonable request.

## Notes

analysis of spin-coated SPION clusters; fabrication of micropatterned iron thin films; proximity analysis of bar-shaped patterns; effects of ROI size and positioning; magnetization of SPIONs and SPION aggregates; magnetometry and MFM signal from iron thin films; parameters for dipole-dipole model (PDF)
