# Supplementary material for: Magnetic Force Microscopy of Micropatterned Clusters of Superparamagnetic Iron Oxide Nanoparticles
Source: ACS Appl Nano Mater. 2025 Jun 5;8(24):12574–82. doi: 10.1021/acsanm.5c01383 (PMC12186231; doi:10.1021/acsanm.5c01383)
Supplement: Supplementary file 1 [file an5c01383_si_001.pdf]

Supporting Information

Supporting Information for "Magnetic Force Microscopy of Micropatterned Clusters of Superparamagnetic Iron Oxide Nanoparticles"

Authors

Kenzington L. Kottenbrock[1], Sierra Reis[2], Gunjan Agarwal[3]*, Samuel D. Oberdick[4,5*]

Affiliations

1. Biomedical Engineering Graduate Program, The Ohio State University, Columbus, Ohio 43210, USA
2. Department of Physics, The Ohio State University, Columbus, Ohio 43120, USA
3. Department of Mechanical and Aerospace Engineering, The Ohio State University, Columbus, Ohio 43210, USA
4. Department of Physics, University of Colorado, Boulder, Colorado 80309, USA
5. National Institute of Standards and Technology, Boulder, Colorado 80305, USA

*agarwal60@osu.edu

*samuel.oberdick@nist.gov

Contents

1. Analysis of Spin-Coated SPION Clusters
2. Micropatterned Iron Thin Films
3. Proximity Analysis of Bar-Shaped Patterns
4. Effects of ROI Size and Positioning
5. Magnetization of SPIONs and SPION Aggregates
6. Magnetometry and MFM Signal from Iron Thin Films

7. Parameters for Dipole-Dipole Model

1. Analysis of Spin-Coated SPION Clusters

SPIONs were spin-coated onto silicon chips, creating heterogeneous clusters averaging 20 nm in thickness. MFM phase images were collected at 60 nm and 100 nm lift heights (Fig. S1b, c). At 60 nm, significant topographical cross-talk can be seen. This artifact spanned the entirety of the SPION clusters and prevented further analysis. At 100 nm, the negative phase signal is very weak but was still detectable. The strength of this signal appeared to be dependent on cluster size (Fig. S1e).

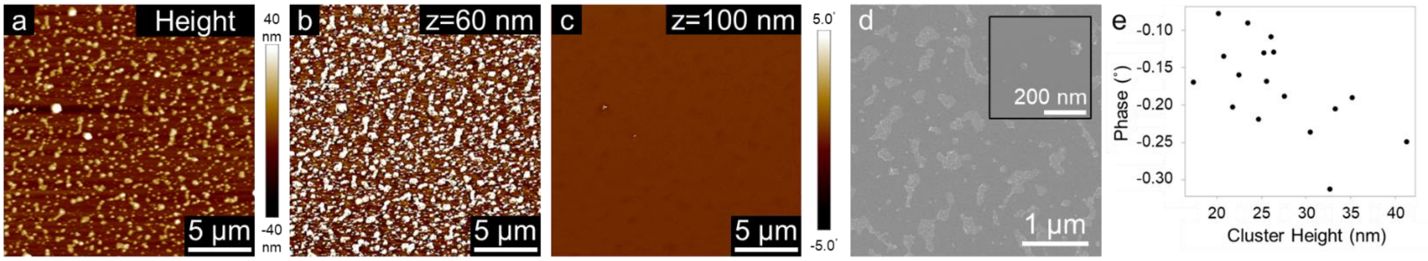

Figure S1: Imaging and analysis of spin-coated SPIONs. (a) Height image and (b,c) corresponding MFM phase images at the indicated lift heights (z). (d) SEM images of spin-coated SPIONs. (e) Plot of MFM phase vs. cluster height.

2. Micropatterned Iron Thin Films

Figure S2 shows a schematic of the fabrication process of patterned iron thin films (a-c) and a scanning electron microscope (SEM) image of the final patterned films (d). The iron films were patterned using the same mask that was used for the SPION-filled wells.

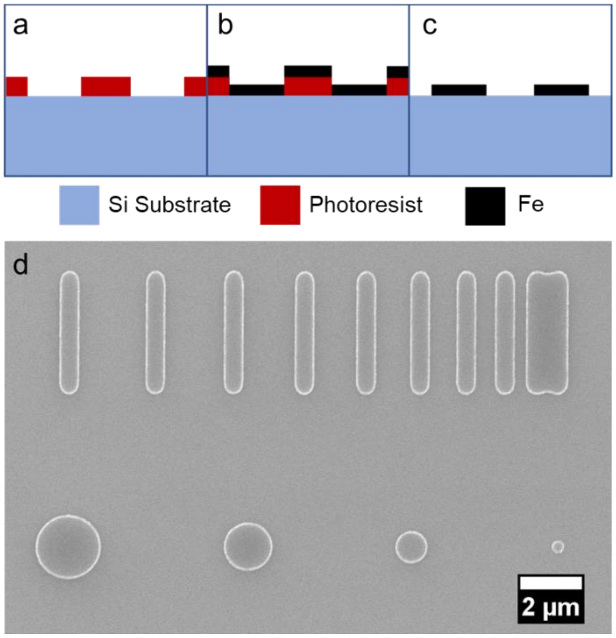

Figure S2: Schematic of the microfabrication procedure used to generate iron thin films: (a) photoresist is patterned, (b) an iron film is evaporated, (c) and the photoresist is removed using a lift-off procedure, leaving a patterned Fe film behind. (d) SEM image of the patterned thin films.

3. Proximity Analysis of Bar-Shaped Patterns

The phase of each bar-shaped pattern was measured and analyzed at a lift height of 100 nm. The spacing between the bar-shaped patterns gradually decreased, allowing for an analysis of pattern proximity. An analysis of variance (ANOVA) test was performed on the MFM data from the SPION-filled wells and the iron thin films. No significant difference was found in the SPION-filled wells, but bar 9 and 1 of the iron thin films had a significantly different phase shift from each other (Fig. S3).

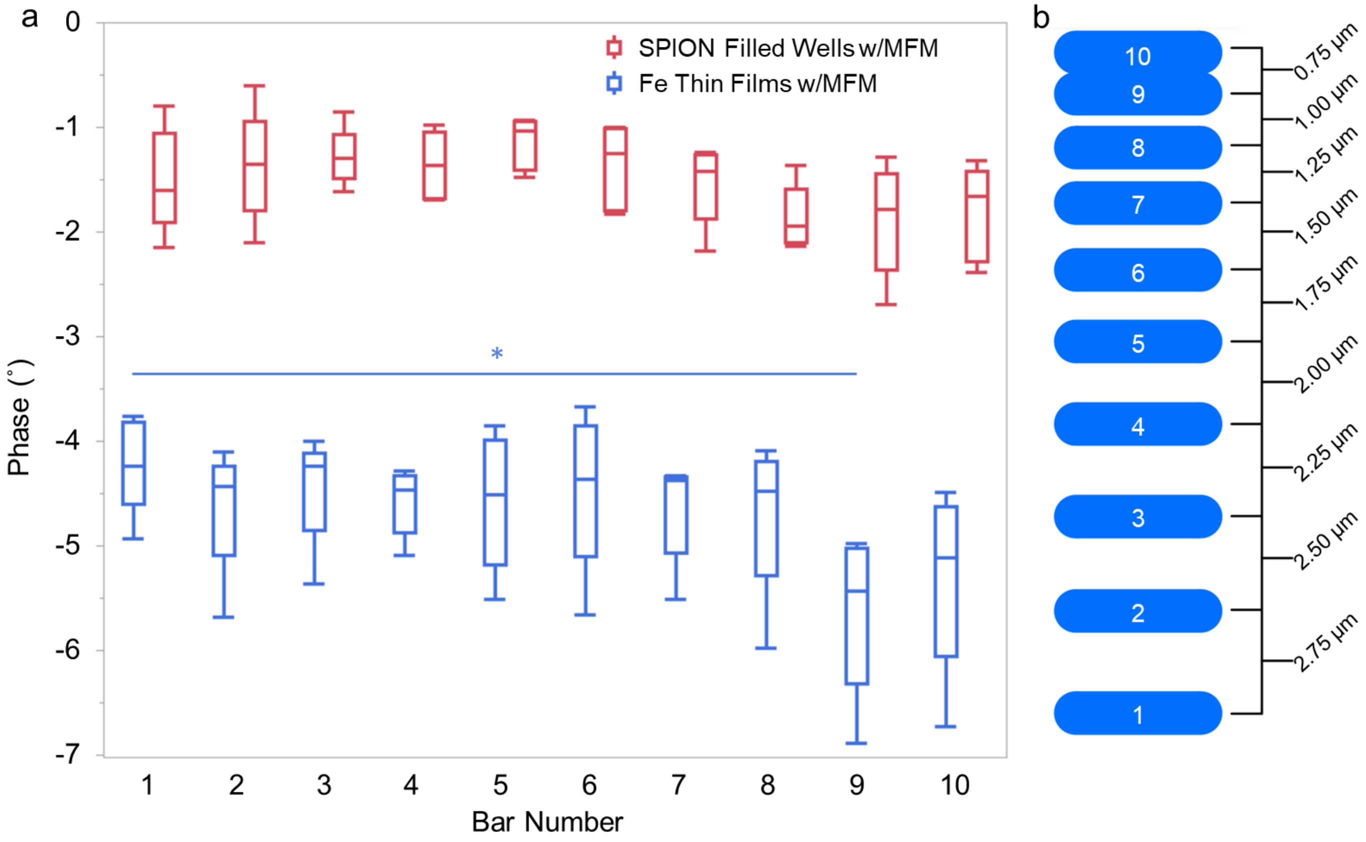

Figure S3: Analysis of the effects of pattern spacing: (a) Box plot showing the phase distribution of each bar shaped pattern at a 100 nm lift height. Bar 9 of the Fe thin films had a significantly different phase from bar 1 ($p < 0.05$). There was no significant difference in the SPION filled bars. (b) Spacing of the bar shaped clusters as measured from their midline.

4. Effects of ROI Size and Positioning

The average phase within the bar-shaped patterns of the empty wells and SPION-filled wells was analyzed using two regions of interest (ROIs): an ROI within the center of the bar (3.98 µm x 0.86 µm) and an ROI containing the bottom half and edge of the bar (3.98 µm x 0.43 µm) (Fig. S4a). The average phase within these ROIs were then plotted as a function of lift height (Fig. S4b). Except for the SPION wells at a lift height of 60 nm, there were negligible differences in the phase collected from each ROI. This is because topographical cross-talk at the edges of the wells created a positive phase shift at 60 nm which affected the average phase of the

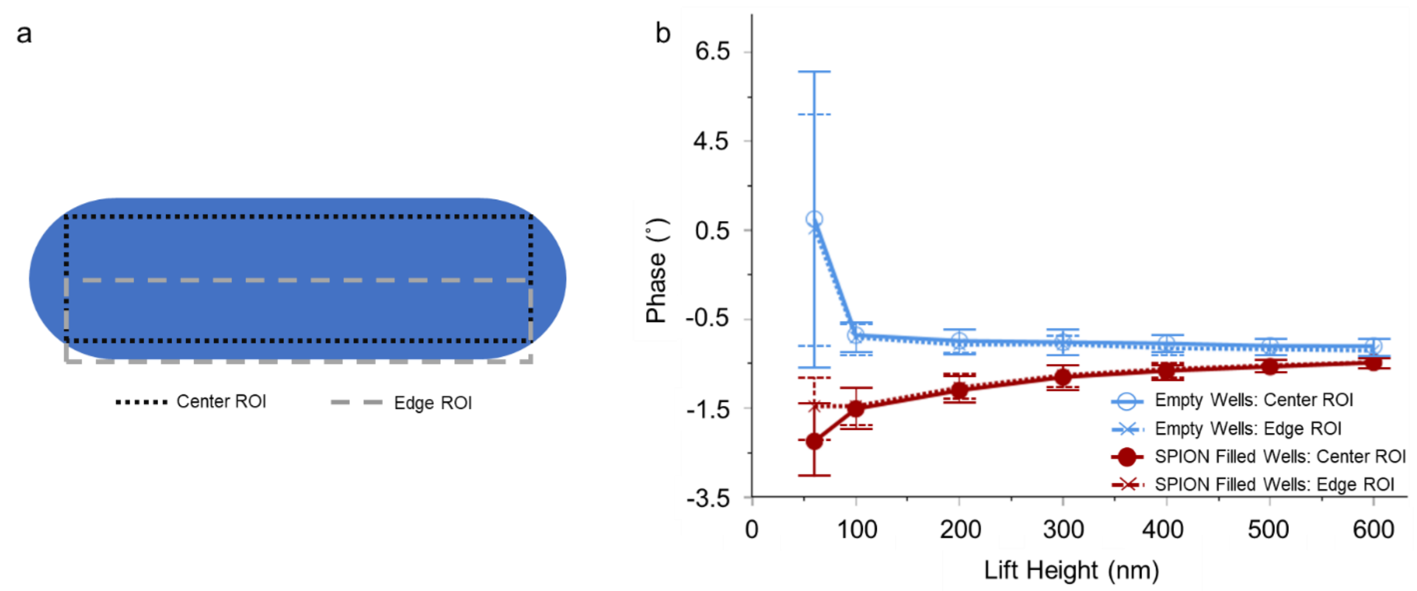

Figure S4. Analysis of the effects of ROI size and position: (a) Schematic of ROI size and position within the bar shaped wells, and (b) the average phase within each ROI as a function of lift height.

SPION-filled wells. In the empty wells, topographical cross-talk affected a large portion of the wells which resulted in a positive phase shift seen in both ROIs.

5. Magnetization of SPIONs and SPION Aggregates

The magnetization of SPION cores was determined experimentally using inductively coupled plasma optical emission spectroscopy (ICP-OES) and magnetometry. Magnetometry was performed using a Quantum Design MPMS 3. ICP-OES measurements were made using a Perkin Elmer Optima 8300 ICP-OES optical system with a segmented-array charge-coupled device detector. The details of the procedure have been previously described in other SPION-related studies[1]. Briefly, a known quantity of SPIONs was digested in nitric acid and the mass of iron in the sample was measured using ICP-OES. Then, another sample with a known mass of iron and corresponding mass of magnetite (Fe_3O_4) was measured with magnetometry to find the saturated magnetic moment at 7 T. SPIONs were embedded in a photocurable polymer to prepare samples for magnetometry. The SPIONs were diluted by a factor of 0.1 in a photocurable hydrogel (0.01 mass fraction of 2,2-dimethoxy-2-phenylacetophenone mixed with poly(ethylene glycol) diacrylate 700, both purchased from Sigma Aldrich). The mixture was cured using ultraviolet light in an environment filled with nitrogen gas. The moment was divided by

the mass of Fe_3O_4 to find the mass magnetization, 54 A·m^2/kg. The mass magnetization was converted to a volume magnetization using the density of Fe_3O_4, ρ = 5240 kg/m^3[2]. The volume magnetization was 283 kA/m.

The volume magnetization of SPION aggregates was approximated by considering the volume fraction of iron oxide cores contained in a random packing of surfactant-coated iron oxide nanoparticles. The volume fraction of randomly packed spheres is $\varphi_{packing} \sim 0.6$[3]. Therefore, within an aggregate of nanoparticles, 60% of the volume will be filled by surfactant-coated iron oxide nanoparticles. The overall volume fraction occupied by iron oxide will be smaller, since some volume is taken up by the surfactant. If we assume a 7 nm iron oxide core diameter with a 1.5 nm thick polymer shell[4], the volume fraction of the iron oxide core in a single surfactant-coated nanoparticle is $\varphi_{core} = 0.343$. The volume magnetization of aggregates was approximated by multiplying the volume magnetization of iron oxide cores by both filling factors, $M_{aggregate} = M_{core} \times \phi_{core} \times \phi_{packing}$ = 58 kA/m.

6. Magnetometry and MFM Signal from Iron Thin Films

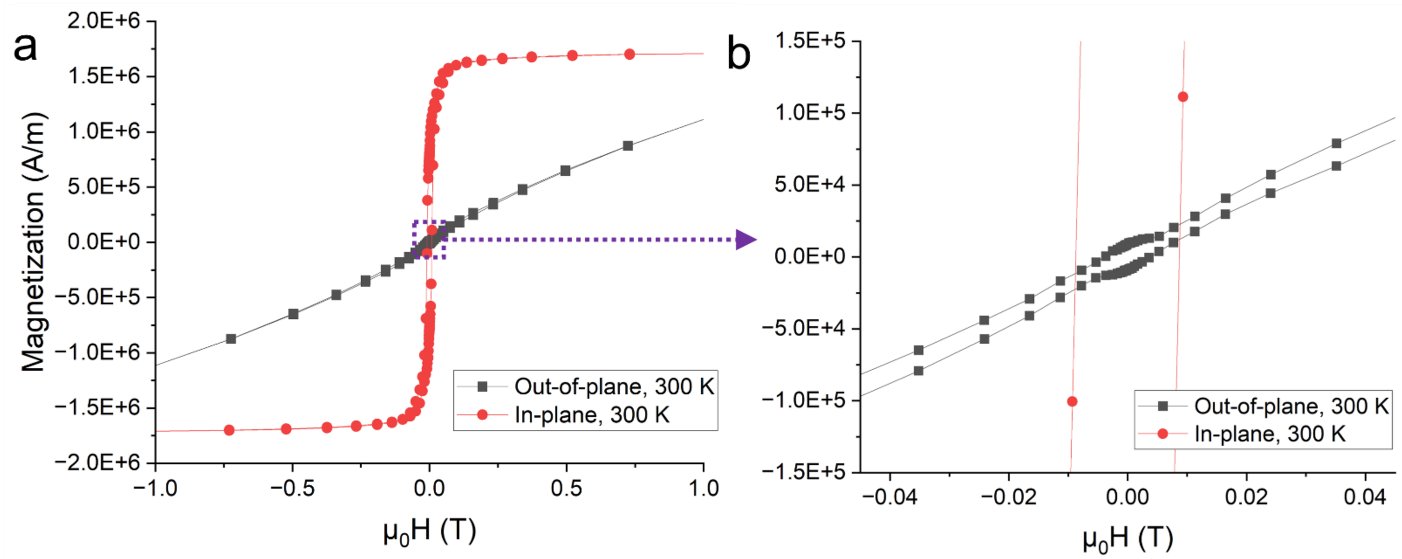

Figure S5. (a) Magnetometry data for the iron micropatterns collected for in-plane and out-of-plane orientations. (b) Magnified region of the graph on the left.

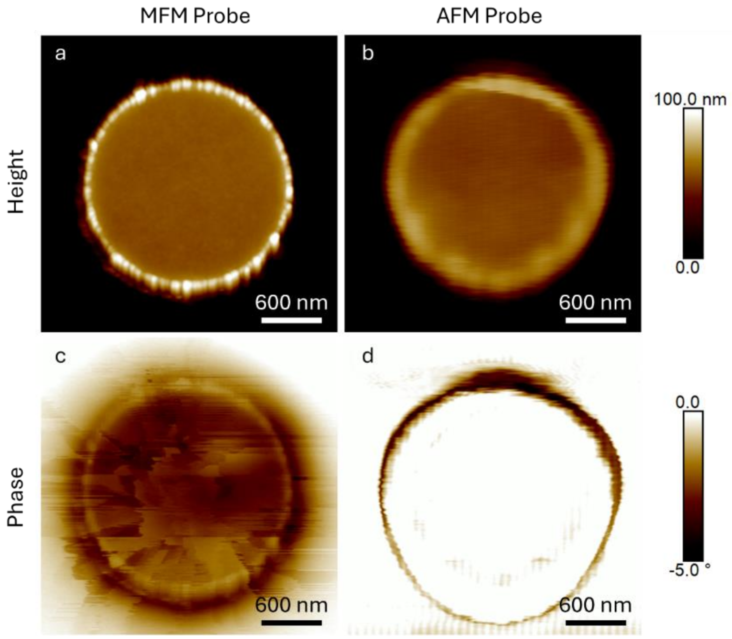

Figure S6. MFM imaging of a 2 μm diameter round iron thin film. Height images of the iron thin film using (a) an MFM probe and (b) an AFM probe and their corresponding phase images at a lift height of 100 nm (c, d).

7. Parameters for Dipole-Dipole Model

Parameter	Value
Spring constant, *k*	2.6
Quality factor, *Q*	160
Standoff for probe tip dipole moment, δ	50 nm
Dipole moment of tip, m_{tip}	3×10^{-16} Am^2
Thickness of interaction volume, *t*	600 nm
Sample magnetization, *M*	58 kA/m
Offset, *C*	-0.47

The parameters were chosen based on experimental details and measurements. The spring constant and magnetic dipole moment for the MFM probe were listed on the supplier's website[5]. The quality factor was evaluated using the system software. The standoff for the probe tip dipole moment was chosen to be half of the thickness of the magnetic coating (100 nm CoCr)[5]. The sample magnetization was calculated from experimental data (see "Magnetization of SPIONs and SPION Aggregates"). The thickness of the interaction volume was set equal to the depth of the micropatterned wells. A constant offset was added to the fit to account for background. The offset, *C*, was chosen to match the phase at the largest lift height (600 nm).

Disclaimers

Any mention of commercial products is intended solely for experimental detail; it does not imply recommendation or endorsement by NIST.

References

[1] Oberdick, S. D.; Jordanova, K. V.; Lundstrom, J. T.; Parigi, G.; Poorman, M. E.; Zabow, G.; Keenan, K. E. Iron oxide nanoparticles as positive T1 contrast agents for low-field magnetic resonance imaging at 64 mT. *Scientific Reports* **2023**, *13* (1), 11520. DOI: 10.1038/s41598-023-38222-6.

[2] Cullity, B. D.; Graham, C. D. *Introduction to magnetic materials*; John Wiley & Sons, 2011.

[3] Scott, G. D.; Kilgour, D. M. The density of random close packing of spheres. *Journal of Physics D: Applied Physics* **1969**, *2* (6), 863. DOI: 10.1088/0022-3727/2/6/311.

[4] Oberdick, S. D.; Borchers, J. A.; Krycka, K. L. Magnetic correlations of iron oxide nanoparticles as probed by polarized SANS in stretched magnetic nanoparticle–elastomer composites. *Applied Physics Letters* **2022**, *120* (5), 052401. DOI: 10.1063/5.0081922.

[5] *ASYMFMHM-R2*. Oxford Instruments. , https://estore.oxinst.com/us/products/afm-probes/afm-probes-catalog/zid805.ASYMFM.HM-R2?srsltid=AfmBOooVO8pj4AVSQevgk9C_lxahhnxqDwIX-9XKdnVHXK5y_c1R0m2W.
